# RSBP: A Reliable Slotted Broadcast Protocol in Wireless Sensor Networks

**DOI:** 10.3390/s121114630

**Published:** 2012-10-31

**Authors:** Phan Van Vinh, Hoon Oh

**Affiliations:** Ubicom Lab, School of Computer Engineering and Information Technology, University of Ulsan, P.O. Box 18, Ulsan 680-749, Korea; E-Mail: pvvinhbk@gmail.com

**Keywords:** RSBP, reliable, broadcast slot, slotted scheduling

## Abstract

In wireless sensor networks for monitoring and control applications, a sink node needs to disseminate messages to all nodes to acquire monitoring data or to control the operation of sensor nodes. The basic flooding protocol suffers from low transmission reliability in broadcasting messages due to the hidden terminal problem. Besides, it can cause the broadcast storm problem by having many nodes rebroadcast the received message simultaneously. In order to resolve these problems while minimizing energy consumption in delivery of broadcast messages, we propose a reliable slotted broadcast protocol (RSBP) that allocates broadcast time slots to nodes based on their slot demands and then allows every node to transmit its broadcast message within the allocated slots. Then, every node can broadcast messages safely in a contention-free manner. Moreover, RSBP can be deployed easily since it does not have any specific requirements such as GPS, multi-channels and directional antennas that may not be always available in real scenarios. We show by experimental study that RSBP significantly outperforms other broadcast protocols in terms of safety-critical packet delivery and energy consumption.

## 1. Introduction

In wireless sensor networks (WSNs) for monitoring and control applications, a sink may collect environmental data periodically and/or send control commands as a need basis to a number of sensor nodes distributed in the factories or the fields. The monitoring data are usually delivered to the sink node along the paths established from the nodes to the sink or using the scheduled slots in TDMA based sensor networks. On the other hand, the sink may have to disseminate some control commands reliably to all nodes in order to control power consumption or request some sensed data in a controlled manner. Especially, in TDMA based sensor networks, the sink needs to send a slot scheduling message and a time synchronization message to all nodes periodically and reliably.

Various approaches have been proposed to apply for broadcast service in WSNs. We can classify the existing approaches into two types: location-based and non-location-based schemes. The location-based approaches require sensor nodes to collect location information of other nodes while the non-location-based ones need to know neighbor information to make retransmission decision. For the location-based schemes [1] and [2], each node and its neighbors exchange location information which is collected by using GPS or other geometric location techniques to compute an optimal local cover set that consists of a minimum number of neighbors to cover hosts within 2-hops. It also requires each node to know its k-hop (k ≥ 2) neighbor information, resulting in high overhead and energy consumption in WSNs. The method proposed in [3] selects a forward node based on probability, and thus it cannot ensure the reliability of the broadcast message transmission. The algorithm in [4] tries to conserve energy by taking advantage of the physical layer design. In [5] and [6], the requirement is that each node must get the geometry information within its 2-hop neighborhood. Maximum Life-time Localized Broadcast (ML^2^B) protocol is proposed in [6] that needs only 1-hop neighbor's information to reduce broadcast overhead. Another paper dealt with an energy issue by using cooperative transmission [7]. In [8] and [9], the Broadcast Protocol for Sensor networks (BPS) exploits the location of each node to broadcast packets in a distributed way. Hence it can reduce the number of redundant rebroadcasts. For the non-location-based schemes, there are also many approaches proposed, including the counter-based approach and probabilistic approaches. In the counter-based approach [3], a node rebroadcasts a packet if the number of duplicate packets received is less than a specified threshold. In the probabilistic approach, a message is rebroadcasted based on probability which can be fixed or calculated dynamically according to node density [10] or neighbor information [11].

In general, most of the previous approaches try to solve the broadcast storm problem by eliminating the rebroadcast of packets based on some information such as location, neighbor information or number of packets received. Actually, there are some approaches that can alleviate the hidden node problem in performing broadcasting, but they require the support of GPS or the use of multi-channels to make localized broadcast decisions. These hardware requirements may not be always available in the real implementation.

Taking into account the problems of broadcast service such as the broadcast storm, hidden terminal, reliability, and energy consumption problem, we introduce an efficient broadcast protocol, called *Reliable Slotted Broadcast Protocol (RSBP)* in which each node is allocated a unique *broadcast time slot* and delivers the broadcast message to its children within the slot. Every node needs to send a demand message which includes a number of broadcast slots required enough for its own and its children's broadcast message delivery. Upon receiving all demands of its children, a sink starts assigning broadcast slots to each of its children. When receiving the assigned slots from the sink, every node allocates broadcast slots for its own children as the sink did. In this way, every node, except for the leaf nodes, receives broadcast slots and can deliver broadcast message to its children without collision or interference since one particular slot is exclusively dedicated to one node. This advantage can solve the hidden terminal problem in broadcast service completely: At any time slot, only one node can send a message, and hence there is no interference or collision during the transmission. The salient features of our proposed RSBP protocol are described as follows:

- High reliability: Data packets can be broadcasted safely to all nodes without collision or interference by using slotted scheduling method and the hidden terminal problem never occurs because only one node transmits a packet in one slot at any time. Comparing with other relative broadcast protocols, RSBP can achieve high packet delivery ratio.
- High energy efficiency: By removing the redundancy of rebroadcast messages, the number of packets that each node has to process is reduced significantly. Moreover, with using slotted scheduling mechanism, it allows sensor node to go to a sleep state after sending or receiving message to conserve energy. The experiment results also show that RSBP outperforms other broadcast algorithms in term of power consumption.
- Low control overhead: Every node only needs to report the demanded number of broadcast slots to its parent and then receive the slot assignment accordingly, thus eliminating the use of other control messages.
- Dynamic and scalability: RSBP can perform well, even in large scale dynamic networks, and a network with the frequent change of topology. As the incoming traffic rate increases, the broadcast slot demand can be updated dynamically to adapt to the increase of bandwidth requirement.
- Easy deployment: it does not need any addition of specific hardware or services such as GPS support, multi-channels or directional antennas. With this advantage, the proposed RSBP protocol can be deployed easily, even in large scale dynamic networks.

The rest of paper is organized as follows: Section 2 describes network model and problem identification. We present the formal description of the proposed protocol in Section 3. Section 4 presents a performance evaluation. Finally, we make concluding remarks in Section 5.

## 2. Preliminary

### 2.1. Network Model

The considered wireless sensor network consists of one sink and many sensor nodes. The radio transmission range of a node is limited for low power consumption and thus a node may reach the other nodes via multiple wireless hops. The nodes form a tree topology originating from a sink node. The sink, wall-powered, acts as monitoring and control server while sensor node equipped with a battery does as data acquisition device. A node senses data from environment periodically or on a need basis and sends it to the sink along tree path. A node can be mobile or immobile, and thus network topology can change dynamically. Two nodes which are located within their mutual radio transmission ranges are said to have a *link*. A link may be broken due to mobility of nodes or some obstacle that intervenes between two neighbor nodes. A node is said to be a *member* if it belongs to a tree. Otherwise it is an *orphan*. A link between a node and its parent is specially called a *tree-link*.

### 2.2. Problem Identification

In this type of network, a sink may have to disseminate control command to the nodes with compound sensor modules in order to request them to turn on/off a specific sensor module to control their power consumption or to the nodes in order to ask them to send at a different rate according to their power levels or the characteristics of data that they acquire. Especially, in TDMA based sensor networks, the sink needs to send a slot scheduling message and a time synchronization message to all nodes periodically. All these commands or messages need to be delivered in a reliable manner.

One simple method is to use a message flooding such that a sink broadcasts a message and upon receiving the message, the other nodes rebroadcast the message only if they have received the message for the first time. However, the flooding scheme incurs the broadcast storm problem that worsens the hidden terminal problem inherently in wireless networks, thus resulting in a lot of collisions. In consequence, this scheme may not be appropriate for the network model that requires the reliability of message transmission.

**Figure 1.**
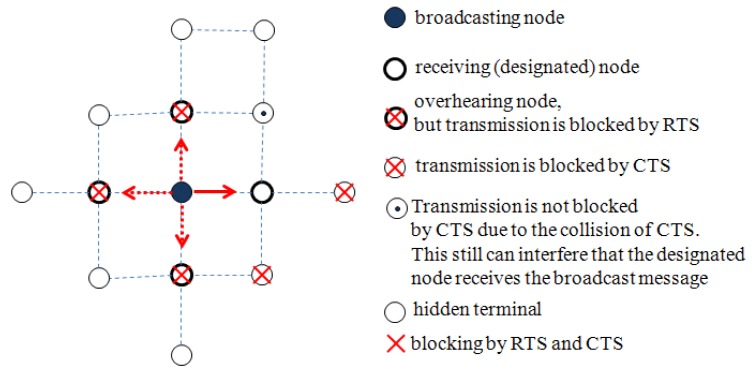
The illustration of the hidden terminal problem.

On the other hand, the RTS/CTS control packets can be employed to provide the transmission reliability of broadcast message, referred to as a unicast-based broadcast (uBroadcast) scheme [20]. This approach can be used when a certain structured topology is maintained such that a node knows its neighbors. A node selects one of its neighbors and sends the broadcast message to the selected or designated node, while the other neighbors overhear the message. It was proven that the uBroadcast contributes to alleviating the hidden terminal problem greatly. The designated node could receive the broadcast message up to almost 100% while the overhearing nodes could receive up to 80%. In this approach, the designated node is only exposed to the hidden terminal problem in a limited way; the overhearing nodes are exposed to the hidden terminal problem freely. Figure 1 illustrates the exposed degree of the hidden terminal problem according to whether it is a designated node or not. Even though the uBroadcast scheme increases the reliability of message transmission, this scheme also may not be appropriate for the applications or the network model that requires a high reliability of message delivery.

The hidden terminal problem is the major cause that hinders the reliable transmission. Since the broadcast message propagates from a sink up to leaf nodes gradually, the hidden terminal problem can be completely removed if we allow a node to issue the message in a controlled manner according to the slots scheduled dynamically by the slot scheduling algorithm. This can be possible in a tree topology in which every node knows its children and its parent. In fact, if we have the number of slots that corresponds to the number of intermediate nodes plus one (for a sink node), the broadcast message can be disseminated to all nodes by having a sink node or each intermediate node broadcast the message within the slot allocated to itself.

## 3. Reliable Slotted Broadcast Protocol (RSBP)

### 3.1. Protocol Structure

**Figure 2.**
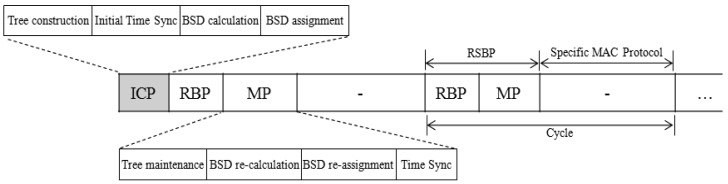
Protocol Structure.

The protocol structure consists of the initial contention period (ICP), and the repeating cycle that includes the reliable broadcast period (RBP), the maintenance period (MP), and the specific MAC protocol period, as shown in Figure 2. In the ICP, a tree is initially constructed and then time synchronization is performed. Furthermore, the broadcast slot scheduling is performed by calculating a broadcast slot demand (BSD) of each node in the tree and assigns the calculated BSD to each node (tree member). ICP is followed by the reliable broadcast period (RBP) and the maintenance period (MP). In the RBP, a broadcast message is disseminated to all nodes using the broadcast slot allocated to each node from the BSD assignment. In MP that follows, the maintenance activities such as tree maintenance, BSD calculation and assignment, and time synchronization are performed in a contention-oriented manner. The following specific MAC protocol period is reserved for the operation of the MAC protocol to be employed. Since the design of a MAC protocol is not a concern of this paper, we do not take into account the specific MAC protocol period in evaluating RSBP in comparison with other protocols.

In fact, the BSDC (BSD calculation) message and the BSDA (BSD assignment) message can be piggybacked in data packet transmitted (from sensor nodes to a sink node) in the specific MAC protocol period or other broadcast messages (e.g., a reliability-critical broadcast message disseminated in RBP), respectively to reduce overhead and delay. Furthermore, the activities in MP are mostly optional. Time synchronization is performed at every k^th^ (k = 8 in this paper) cycle and the other activities are performed conditionally if necessary.

### 3.2. Tree Construction and Maintenance

A tree is constructed originating from a sink while sensor nodes synchronize their time in the process of tree construction. A sink as the only member at the beginning initiates a tree construction process by broadcasting an advertisement (ADV) message. Upon receiving the ADV message, an orphan node joins the member by sending a join request (JREQ) message to the member. If a member receives JREQ, it takes the orphan node as its child and replies with a join response (JRES) message. Upon receiving JRES from the member, the orphan node becomes the child of the member. In addition, another orphan who has overheard the JREQ can take the same procedure to become a member. If an orphan overhears the JREQ messages from multiple members, it pairs with a member that has the shortest distance to the sink.

Tree maintenance is performed during the MP sub-period of the RSBP period. The tree is maintained dynamically for the broken part of the tree. If a certain node detects the failure of the link to its parent, it tries to find one neighbor that provides the shortest distance to a sink and then joins the node by sending JREQ. In this case, BSDs of the nodes related to the broken link have to be changed accordingly. Thus, the parent of the detecting node has to issue the BSDC_MSG to its parent while the detecting node has to issue the BSDC_MSG to its new parent. If a sink node receives the BSDC_MSG, it performs the BSD assignment algorithm.

### 3.3. Broadcast Slot Demand (BSD) Calculation Algorithm

When the tree construction phase is complete, every node enters the slot scheduling phase to assign time slots for delivery of broadcast messages. Firstly, every node needs to collect all its children's slot demands. Starting with the leaf nodes, every node sends a slot demand message to its parent including the number of broadcast slots enough to deliver its broadcast message to all its descendants. After receiving all slot demand messages from its children, a node performs the slotted scheduling algorithm to assign time slots to its children by sending a slot scheduling message, starting from the sink and proceeding down to leaf nodes recursively.

Starting with a leaf node, a node *i* sends a *broadcast slot demand calculation* (*BSDC*) message represented as BSDC_MSG = (*τ_i_*) where *τ_i_* corresponds to the number of nodes that needs a broadcast time slot in the subtree whose root is itself, to its parent. The formula to calculate *τ_i_* is given as follows:

(1) $$\begin{array}{c} \tau_{i}=\{\begin{array}{cc} \sum_{k\in \mathrm{ch}(i)}\tau_{k}+1 & \left(\text{if node i is not a leaf node}\right)\\ 0 & \left(\text{if node i is a leaf node}\right) \end{array}\\ \text{where ch i is a children set of node i} \end{array}$$

Each node *i* calculates *τ_i_* based on Equation (1). Then, BSD(s) (=*τ_s_*) for a sink node *s* indicates the total number of broadcast slots for a reliable dissemination of a broadcast message to all nodes. Since this function is recursive, the BSD of each node should be calculated progressively, starting from the leaf nodes and moving up to a sink node.

**Figure 3.**
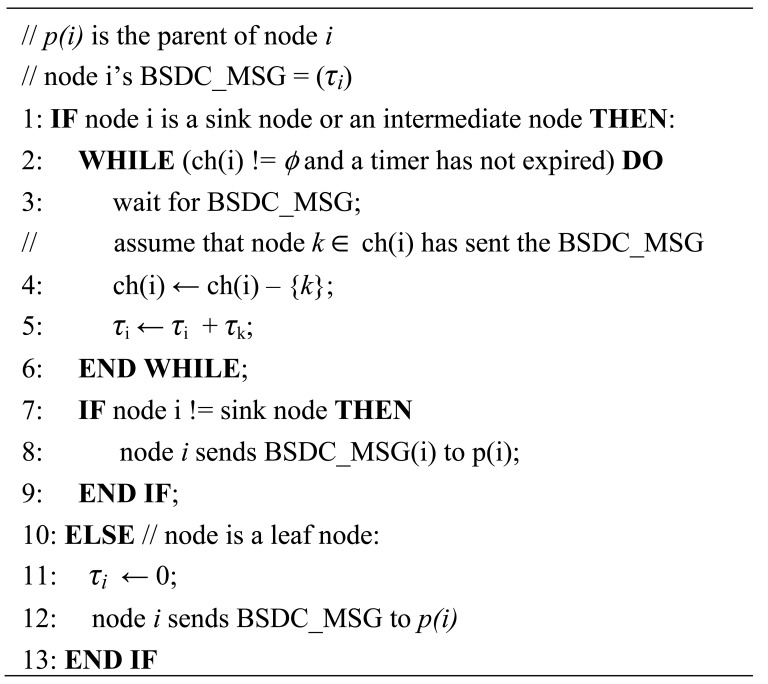
BSD calculation algorithm.

Figure 3 shows the BSD calculation algorithm in which every node except for the sink sends one BSD message. Thus, the complexity of message transmission of the BSD algorithm is given *O(n)*, *n* is the number of nodes. A timer is used to prevent the locking of the distributed algorithm.

### 3.4. Broadcast Slot Demand (BSD) Assignment Algorithm

The broadcast slots are assigned to each node according to the BSDs which was calculated by the BSD calculation algorithm. The BSD assignment starts from the sink and proceeds down to leaf nodes recursively. The BSD assignment algorithm employs a new message *BSDA_MSG*.

Each node *i* sends *BSDA_MSG = (startSlot(i), timeStamp)* to its children to notify the start slot position of each child and *timeStamp* for time synchronization, where startSlot(i) is an array of c_ij_ = (j, startPos) that indicates each child j and its start slot position with respect to the BSD(s). In this way, each node *i* gets to know and manages its BSD(i) (which was calculated by the BSD calculation algorithm) from its start slot position. Node *i* uses the first slot of BSD(i) starting from its start slot position as its broadcasting slot and then schedules the remaining slots (BSD(i) – 1) to its own children in the same way.

**Figure 4.**
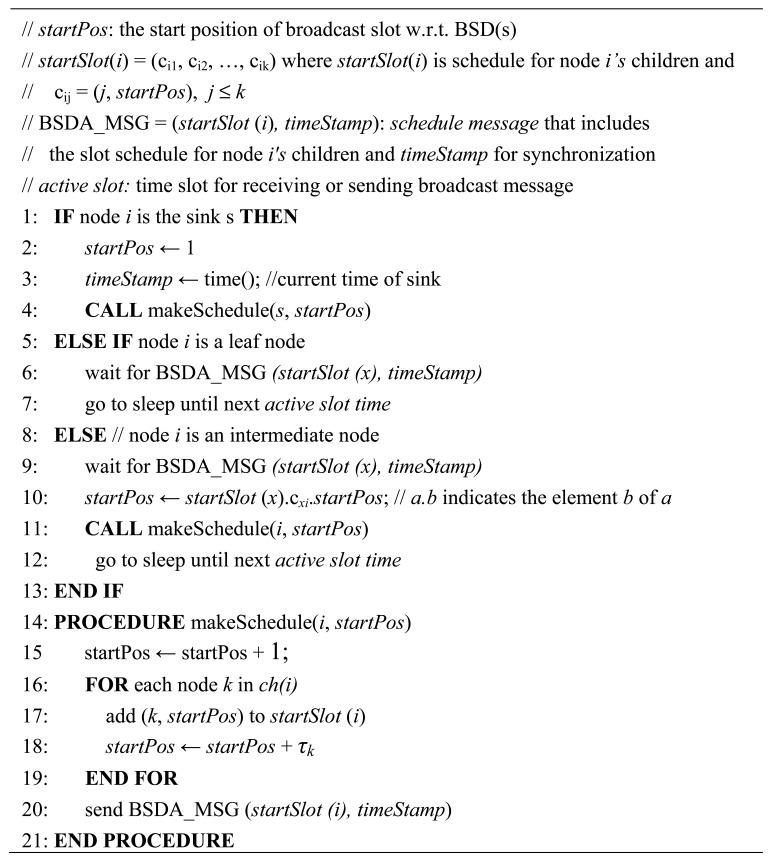
BSD assignment algorithm.

Figure 4 details the BSD assignment algorithm. The complexity of message transmission of the BSD assignment algorithm is given *O*(*n*), *n* is the number of intermediate nodes that needs to send *BSDA_MSG* to its children.

**Figure 5.**
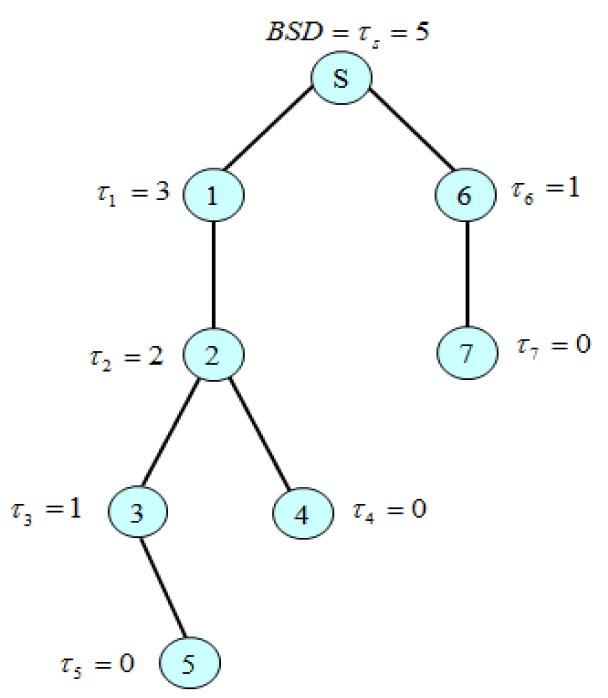
Example of BSD calculation.

**Figure 6.**
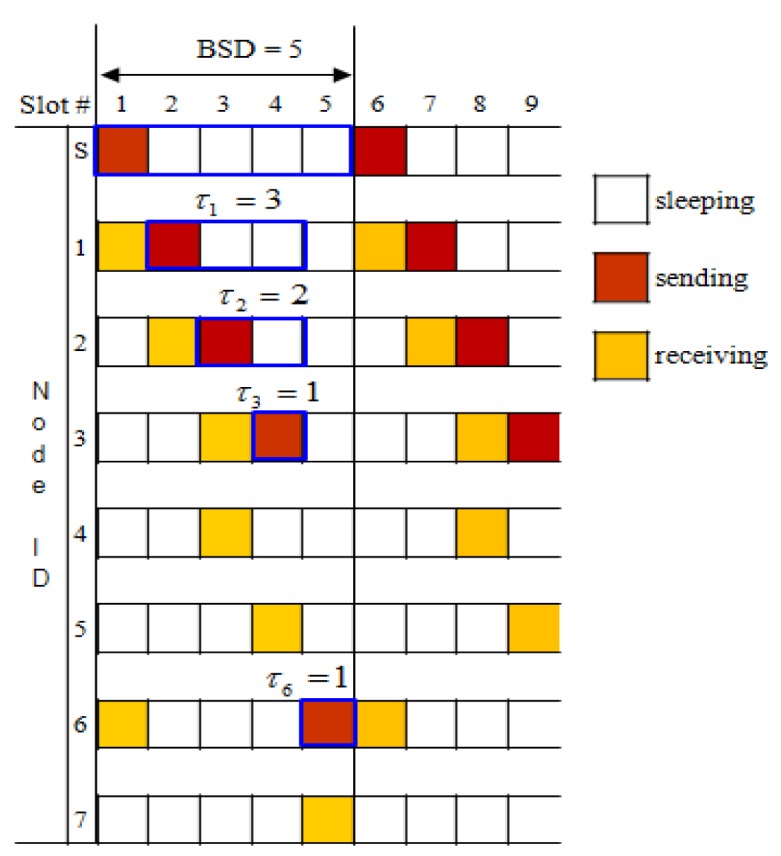
Example of BSD assignment.

Figure 5 shows an example of the BSD calculation. Leaf nodes do not have to rebroadcast a message: *τ_4_* = *τ_5_* = *τ_7_* =0. Since *τ_1_* = 3 and *τ_6_* = 1, BSD(s) = *τ_s_* = 5. Figure 6 shows an example of the BSD assignment for the simple tree shown in Figure 5. The sink *s* uses the first slot as its broadcasting slot and notify (1, 2) and (6, 5) (= (child id, start position)) according to the line 17 of the BSD assignment algorithm by sending *BSDA_MSG* to its children 1 and 6. Then, receiving children 1 and 6 take the starting slot 2 and the starting slot 6 as its broadcasting slot, respectively as illustrated in Figure 6.

In this way, every node knows a broadcast slot to deliver the message. Therefore, from the next cycle, each node can broadcast the received message at the allocated broadcast slot time. Note that the BSDA message can be piggybacked in a broadcast message to reduce overhead. In addition, the RSBP reduces power consumption by managing the active and sleep times. In every cycle, each node can get into a sleep state immediately after receiving or sending a message until the next active slot time (*active slot* is a time slot for sending or receiving a message).

### 3.5. Time Synchronization

As a TDMA-based mechanism, RSBP requires network time synchronization to perform its operation accurately. Time synchronization is performed during the MP sub-period of the RSBP period at the multiple of *k* cycles. There are some existing solutions for time synchronization [17–19] that can be applied to WSNs. In the RSBP protocol, we employ the Flooding Time Synchronization Protocol (FTSP) [18], a multi-hop time synchronization protocol, which achieves high precision performance by utilizing MAC layer time stamping and comprehensive error compensation including linear regression for clock skew estimation.

The synchronization message (*SYNC*) contains four fields (*rootID, nodeID, seqNum, globalTime*): The *rootID* is the ID of a root node, the *nodeID* is the ID of a sender for maintaining neighbor relationship, the *seqNum* is the sequence number generated by the root node which is used to handle redundant synchronization messages, and the *globalTime* is the current time of the root that is estimated by a transmitter when *SYNC* is broadcast. The sink that has the lowest ID (*nodeID = 0*) becomes a root. The root generates *SYNC* every *k* cycles. Upon receiving *SYNC*, the receiver obtains the local time that refers to the same instant as global time in *SYNC* from the viewpoint of the receiver's local clock. Therefore, *SYNC* provides a synchronization point being a pair <global time, local time> for each receiver. The difference between the global time and the local time of a synchronization point becomes the clock offset of the receiver and the root. However, the offset is not constant due to clock drift, so linear regression is used to compensate for clock drift. Each node maintains the regression table including eight data points where each data point is a pair of the offset and the local time that is updated upon receiving a new SYNC message. Applying the linear regression method for two independent variables, *offset* and local time (*LT*), the linear equation for the regression of *offset* on *LT* can be given as follows:

(2) $$\text{offset}=\bar{\text{offset}}+\text{skew}*(\text{LT}-\bar{\text{LT})}$$

where $\text{skew}=\frac{\overset{n}{\underset{i=1}{\text{∑}}}\left({\text{LT}}_{i}-\bar{\text{LT}})({\text{offset}}_{i}-\bar{\text{offset}}\right)}{\overset{n}{\underset{i=1}{\text{∑}}}{\left({\text{LT}}_{i}-\bar{\text{LT}}\right)}^{2}}$, $\bar{\text{LT}}=\frac{1}{n}\overset{n}{\underset{i=1}{\text{∑}}}{\text{LT}}_{i}$, $\bar{\text{offset}}=\frac{1}{n}\overset{n}{\underset{i=1}{\text{∑}}}{\text{offset}}_{i}$, and *n* is set to 8.

Therefore, given *LT*, each node can estimate the *global time (GT)* of the root by:

(3) $$\text{GT}=\text{LT}+\bar{\text{offset}}+\text{skew}*(\text{LT}-\bar{\text{LT})}$$

In the original FTSP, *SYNC* is broadcast periodically from a root node and flooded down to the leaf nodes. In our implementation, only a node that has at least one child re-broadcasts SYNC.

## 4. Performance Evaluation

### 4.1. Experiment Setup

To evaluate performance of the RSBP protocol, we conducted an experiment with an indoor Testbed of TelosB motes running TinyOS 2.1. The TelosB mote uses Chipcon CC2420 radio which is compliant with the IEEE 802.15.4 PHY layer standard in the 2.4 GHz ISM band with an effective data rate of 250 kbps [16]. The mote uses an 8 MHz TI MSP430 microcontroller with 10 kB RAM. The current draw of TelosB, excluding the radio, is 1.8 mA in active mode and 5.1 μA when in sleep mode. The CC2420 radio consumes 23 mA in receiving/listening mode, 17.4 mA when transmitting at 0 dBm, 21 μA in idle mode, and 1 μA in sleep mode. We can see that the power consumption of the radio when receiving/transmitting is much greater than that when in sleep mode. Therefore, to extend network lifetime, the radio should be turned on and off according to the duty cycle and the time when a node operates in receiving/transmitting or listening mode should be kept as less as possible.

In our experiment, motes are powered using batteries, except for the sink which is powered by a USB interface connected to a computer. Sensor nodes are uniformly distributed in an office with dimensions of 20 m × 30 m. The sink node is placed at the top center of the experimental area. When the number of nodes increases, the tree size also increases proportionally. The transmitting power level is set to level 3 (−25 dBm) and the corresponding radio transmission range is 5 m, approximately. Some key parameters in the experiment are shown in Table 1.

Since our proposed protocol does not use location information that needs GPS support, RSBP is compared with other non-location-based protocols such as the basic IEEE 802.11 flooding and the dynamic probabilistic flooding algorithm based on neighbor information (abbreviated as DPFA) [11]. DPFA allows sensor nodes to rebroadcast a packet according to their retransmission probability that is calculated based on the numbers of child and sibling nodes. In [11], DPFA showed that it performs better than other methods (such as flooding, fixed probabilistic flooding and dynamic probabilistic flooding in [10]) in terms of packet delivery ratio and the number of retransmissions. We compare RSBP with the basic flooding and the DPFA protocol using the following performance metrics:

- Packet delivery ratio (PDR): The ratio of the average number of nodes that has received the broadcast message issued by a sink to the total number of nodes in the network.
- Retransmission ratio: The ratio of the average number of nodes that has rebroadcasted packet to the number of nodes that has received broadcast message in the entire network.
- Packet processing load: The average number of packets that each node has to process (transmit or receive) for one packet issued by a sink. Note that a node may receive multiple copies of a packet, leading to increasing the packet processing load.
- Active time ratio: The ratio of time that each node remains active to process the broadcast messages to the total experiment time.
- End-to-end delay: the elapsed time from the time when the sink broadcasts a message until the time when the last node on the network receives the message.

### 4.2. Experimental Results

#### 4.2.1. Packet Delivery Ratio

Packet delivery ratio (PDR) is one of the most important performance metrics in design of a reliable broadcast protocol that aims at delivering packets safely from a sink to all nodes in the network.

**Figure 7.**
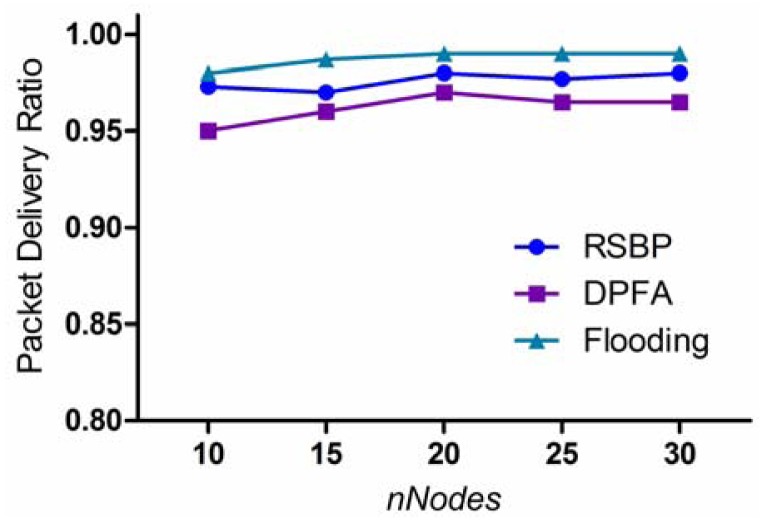
Packet delivery ratio.

Figure 7 shows PDR according to variation of *nNodes* (the number of nodes). We can see that three protocols achieve good results, greater than 95% in all cases. The basic flooding protocol achieves the best performance in PDR, 99% on an average. However, it has to pay a high cost in retransmission ratio and processing load compared to the other broadcast protocols. DPFA proposed in [11] can achieve a good PDR, 96% on an average. In DPFA, a node can adjust the retransmission probability dynamically according to the number of its children and sibling nodes. Thus, it achieves a good PDR while reducing the number of retransmissions. However, its performance is lower than the other two's because it does not handle the hidden terminal problem effectively.

It is worth noting that RSBP fails to achieve 100% of delivery ratio. This is because only one node sends the broadcast message to its children, and thus the message may not be delivered successfully to some distant child due to signal attenuation. However, with the flooding scheme, different nodes issue the broadcast message toward a particular node. This will increase the probability of reception, even though there does exist signal attenuation problem.

#### 4.2.2. Retransmission Ratio

According to the previous evaluation, the flooding algorithm can achieve the best PDR, but suffers from the high retransmission cost. Indeed, in the flooding protocol, every node retransmits the packets that it received, leading to the increased number of duplicate packets. This causes the broadcast storm problem and wastes energy. To address this issue, in this section, we evaluate three protocols comparatively in terms of retransmission ratio.

**Figure 8.**
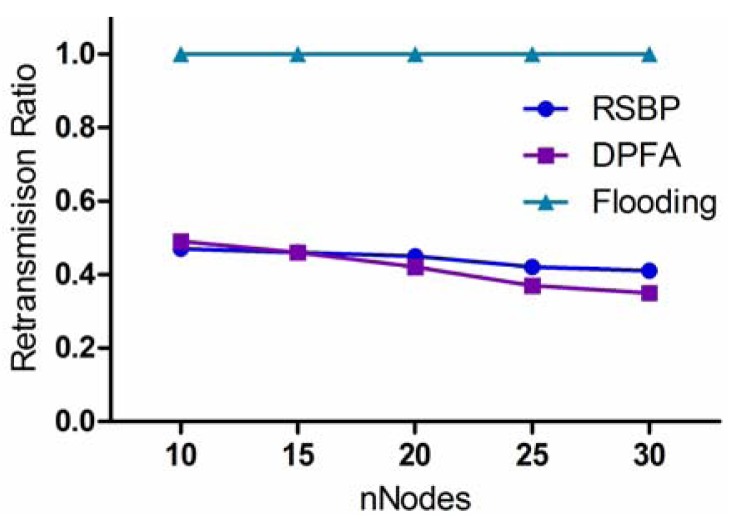
Retransmission ratio.

Figure 8 compares the retransmission ratios of the three protocols. The transmission ratio of the flooding protocol is always 1.0 because it requires every node to retransmit a received packet. Whereas, RSBP and DPFA show good performance in retransmission ratio, less than 0.45, and they have a slightly decreasing curve as *nNodes* increases. For RSBP, when receiving a packet, only the node which has children will rebroadcast a packet to its children within the assigned slot. This helps RSBP reduce the number of packet retransmissions while achieving a good PDR. Similarly, in DPFA, each node rebroadcasts a packet according to its retransmission probability which depends on the number of its children and sibling nodes. In this way, DPFA can reduce the retransmission ratio, but does not make sure that the packet is delivered safely to all nodes in the network. From Figure 8, even though DPFA is slightly better than RSBP in retransmission ratio, it does not mean that DPFA has lower power consumption than RSBP because energy is mostly consumed when a node transmits or receives a packet, or stays in idle mode. Therefore, to evaluate the performance of three protocols precisely in power consumption, we will compare them in terms of packet processing load and active time ratio.

#### 4.2.3. Packet Processing Load

Because the energy resources of sensor nodes are limited, reducing energy consumption is of great importance in WSNs. Our goal is to design a reliable broadcast protocol with low power consumption, by reducing the number of packets that a node has to transmit or receive as much as possible. To evaluate performance of the RSBP protocol about energy consumption, we compare our protocol with the others in terms of packet processing load.

**Figure 9.**
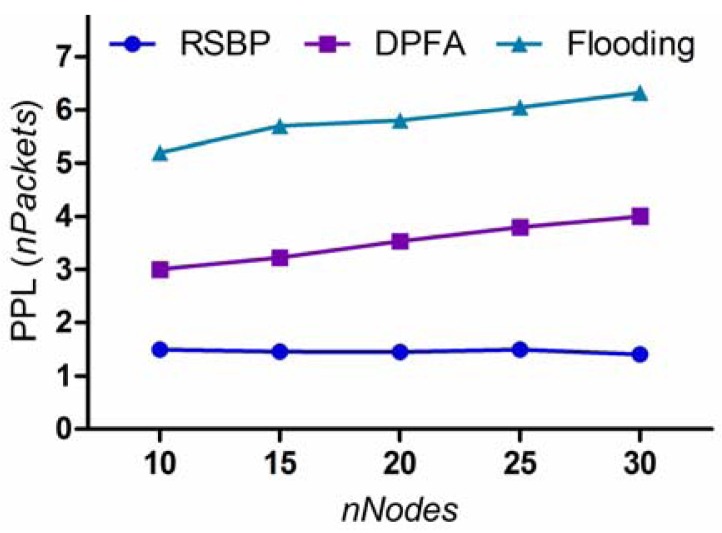
Packet processing load (PPL).

As shown in Figure 9, the packet processing load of the RSBP protocol is much smaller than those of DPFA and Flooding, around 1.5 packets on an average. In RSBP, every node works in a duty cycle: An active state in receiving and sending slots, and a sleep state at other slots. Thus, each node receives only one packet and rebroadcasts this packet only if it has children. Whereas, the packet processing loads of DPFA and Flooding are so high, about 2 or 3 times higher than that of RSBP, and tend to increase steadily in proportion to the number of nodes. It means that, in DPFA and Flooding, a node may receive a lot of redundant packets, especially in a dense network, leading to wasting energy resource and degrading performance significantly. Moreover, it is also shown in Figure 9 that RSBP reduces the packet processing load about 75% and 60% compared with Flooding and DPFA, respectively.

#### 4.2.4. Active Time Ratio

One more metric that can be used to evaluate the energy consumption is *active time ratio*, which is defined as the ratio of active time of each node to the total experiment time. Notice that RSBP using the slotted scheduling method allows sensor nodes to go to a sleep state for the purpose of energy saving.

**Figure 10.**
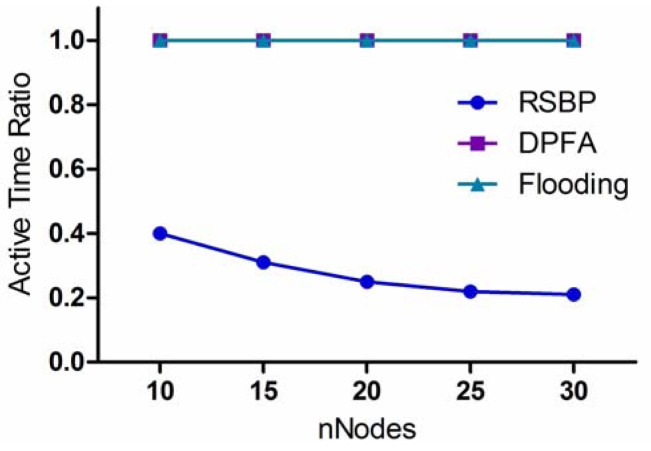
Active Time Ratio.

Figure 10 shows the comparison of the three protocols in terms of *active time ratio* according to variation of *nNodes*. As we can see, the active time of each node in RSBP is much less than those of the other two protocols because in RSBP, sensor nodes have a sleep and active mode while in both DPFA and Flooding, they always stay in an active state for processing packets during the operating time. In addition, it is also shown in Figure 10 that the active time ratio of RSBP decreases gradually, reaching less than 0.25 when the number of nodes is over 20. The total broadcast slots increase according to the increase in the number of nodes, leading to the decrease in active time ratio. The results of packet processing load and active time ratio prove that the RSBP protocol is an energy-efficient broadcast protocol which prolongs network lifetime.

#### 4.2.5. End-to-End Delay

**Figure 11.**
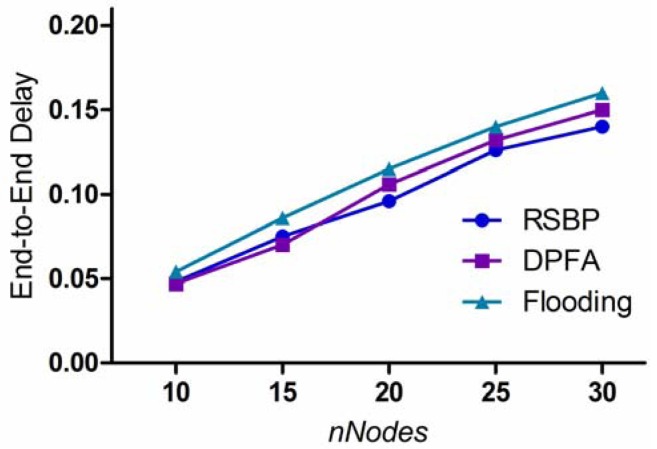
End-to-End Delay.

In order to measure *end-to-end delay (E2E)* more accurately, we also implemented the time synchronization protocol for DPFA and the flooding algorithm. Figure 11 compares the three protocols by varying *nNodes* in the dimension of 20 m × 30 m. It is shown that the three protocols have an increasing E2E curve in common as node density increases. In RSBP, E2E depends on the size of BSD. If *nNodes* increases, the number of nodes that will be involved in the broadcast activity will obviously increase, thus resulting in the increasing E2E curve. On the other hand, Flooding and DPFA will experience more collision and interference as *nNodes* increase because of the use of contention-based channel access. This is why these two also show increasing curves. Comparing RSBP and the others, the figure indicates that collisions are more sensitive to the delay than the increase in the size of BSD.

### 4.3. Performance Metric Analysis

In Section 4.2, we used some performance metrics to evaluate RSBP. Since every node works in consecutive cycles, we can estimate the performance metrics of the RSBP protocol. It is assumed that each node requires at most one broadcast slot to rebroadcast a packet in one cycle. We use *n* as the total number of nodes, except for a sink and *BSD* as the total number of broadcast slots.

#### Packet Delivery Ratio (PDR)

In RSBP, each node is allocated one unique slot for broadcasting a packet safely without collision. Therefore, the RSBP protocol can achieve the performance of *PDR* ≈ 1.

#### Retransmission Ratio (*ReRatio*)

In RSBP, only the intermediate nodes that have children except for a sink node and leaf nodes (= BSD) rebroadcast a receiving message. Thus, *ReRatio* is given as follows:

ReRatio=BSD/n<1

#### Packet Processing Load (*PPL*)

In every cycle, the intermediate nodes (= BSD) process two messages (one receiving and one transmitting) and (n—BSD) nodes either transmit (one sink) or receive (leaf nodes) a broadcast message. The packet processing load is given as follows:

PPL=((2×BSD)+(n−BSD))/n=1+BSD/n

#### Active Time Ratio (*ActRatio*)

We have the BSD nodes that need two broadcast slots (one receiving slot and one sending slot) and (n—BSD) nodes that need only one receiving slot. Thus, Active Time Ratio can be calculated as follows:

ActRatio=((2×BSD)+(n−BSD))/(n×BSD)=1/n+1/BSD

We can see that these performance metrics depend on the number of nodes *n* and total number of broadcast slots *BSD*. For example, with the same number of nodes *n*, we can get better *ReRatio* and *PPL* by reducing *BSD*. We can achieve this by reducing depth or increasing the radio transmission range.

#### End-to-End Delay (E2E)

In RSBP, each intermediate node is allocated one unique slot for broadcasting a packet and the total number of broadcast slots required is BSD. Thus, *E2E* = *BSD.*

## 5. Concluding Remarks

In this paper we have proposed an efficient broadcast protocol that not only delivers broadcast message reliably to all nodes in the network, but also reduces energy consumption significantly. By using the slotted scheduling algorithm, RSBP efficiently resolves the hidden terminal problem, broadcast storm problem and energy constraint problem in WSNs. Another advantage of RSBP is that it does not have any specific requirements such as GPS, multi-channels, and directional antennas which may not be always available in the practical environment. In the experimental results, we show that RSBP outperforms the other broadcast methods in terms of safety-critical packet delivery and energy consumption, and complements the shortcomings of the other broadcast algorithms. To the best of our knowledge, RSBP is the first efficient broadcast protocol using a slotted scheduling mechanism that can transmit a broadcast message reliably to all nodes, achieving low power consumption.

## Figures and Tables

**Table 1.** RSBP experiment parameters.

**Parameter**	**Value**
Default transmission power	−25 dBm (power level 3)
Channel frequency	2.480 MHz (channel 26)
Sensor model	TelosB
Slot size	12 ms
Data packet length	80 bytes
Dimensions	20 × 30 (m^2^)
Number of nodes	1 sink; 30 sensor nodes
Experiment time	3,600 s
